# Efficient gene-driven germ-line point mutagenesis of C57BL/6J mice

**DOI:** 10.1186/1471-2164-6-164

**Published:** 2005-11-21

**Authors:** Edward J Michaud, Cymbeline T Culiat, Mitchell L Klebig, Paul E Barker, KT Cain, Debra J Carpenter, Lori L Easter, Carmen M Foster, Alysyn W Gardner, ZY Guo, Kay J Houser, Lori A Hughes, Marilyn K Kerley, Zhaowei Liu, Robert E Olszewski, Irina Pinn, Ginger D Shaw, Sarah G Shinpock, Ann M Wymore, Eugene M Rinchik, Dabney K Johnson

**Affiliations:** 1 Life Sciences Division, Oak Ridge National Laboratory, P.O. Box 2008, Oak Ridge, TN 37831, USA; 2 The University of Tennessee-Oak Ridge National Laboratory Graduate School of Genome Science and Technology, Oak Ridge, TN 37830, USA; 3 Department of Biochemistry, Cellular, and Molecular Biology, The University of Tennessee, Knoxville, TN 37996, USA; 4 SpectruMedix, 2124 Old Gatesburg Road, State College, PA 16803, USA; 5 Taconic, 273 Hover Avenue, Germantown, NY 12526, USA

## Abstract

**Background:**

Analysis of an allelic series of point mutations in a gene, generated by *N*-ethyl-*N*-nitrosourea (ENU) mutagenesis, is a valuable method for discovering the full scope of its biological function. Here we present an efficient gene-driven approach for identifying ENU-induced point mutations in any gene in C57BL/6J mice. The advantage of such an approach is that it allows one to select any gene of interest in the mouse genome and to go directly from DNA sequence to mutant mice.

**Results:**

We produced the Cryopreserved Mutant Mouse Bank (CMMB), which is an archive of DNA, cDNA, tissues, and sperm from 4,000 G_1 _male offspring of ENU-treated C57BL/6J males mated to untreated C57BL/6J females. Each mouse in the CMMB carries a large number of random heterozygous point mutations throughout the genome. High-throughput Temperature Gradient Capillary Electrophoresis (TGCE) was employed to perform a 32-Mbp sequence-driven screen for mutations in 38 PCR amplicons from 11 genes in DNA and/or cDNA from the CMMB mice. DNA sequence analysis of heteroduplex-forming amplicons identified by TGCE revealed 22 mutations in 10 genes for an overall mutation frequency of 1 in 1.45 Mbp. All 22 mutations are single base pair substitutions, and nine of them (41%) result in nonconservative amino acid substitutions. Intracytoplasmic sperm injection (ICSI) of cryopreserved spermatozoa into B6D2F1 or C57BL/6J ova was used to recover mutant mice for nine of the mutations to date.

**Conclusions:**

The inbred C57BL/6J CMMB, together with TGCE mutation screening and ICSI for the recovery of mutant mice, represents a valuable gene-driven approach for the functional annotation of the mammalian genome and for the generation of mouse models of human genetic diseases. The ability of ENU to induce mutations that cause various types of changes in proteins will provide additional insights into the functions of mammalian proteins that may not be detectable by knockout mutations.

## Background

A major challenge following the sequencing of the human genome is to determine the biological functions of the estimated 30,000 genes. Inducing mutations in mouse genes and determining their consequences in the whole animal is a powerful approach for gaining insight into the functions, regulatory networks, and gene-environment interactions of homologous human genes. To provide a systematic and comprehensive functional annotation of every gene in the genome using mouse mutagenesis will undoubtedly require numerous complementary strategies, such as gene knockouts, conditional knockouts, and point mutagenesis with the chemical *N*-ethyl-*N*-nitrosourea (ENU) [1,2].

The ethylating chemical ENU is the most potent mutagen in the mouse, with a per-locus mutation frequency, based mainly on detecting mutant phenotypes at visibly marked loci, ranging from approximately 1/1500 for a single 250 mg/kg dose to 1/700 for a fractionated 4 × 100 mg/kg dose [3,4]. Since ENU induces primarily single base pair (bp) substitutions in DNA, it is especially useful for producing an allelic series of mutations in a gene, where each mutation may result in different degrees of severity of the mutant phenotype or even in completely different mutant phenotypes. Depending on the location of the mutation within the gene and on the specific base pair substitution, ENU-induced mutations may cause amorphic (loss of function), hypomorphic (partial loss of function), antimorphic (opposing/dominant negative function), hypermorphic (exaggerated function), and neomorphic (novel gain of function) protein changes, which permit a fine-scale dissection of gene function and generally reflect the types of gene variations found in the human population. This ability of ENU to induce mutations that cause various types of changes in proteins provides valuable insights into protein structure and function that cannot be obtained with knockout mutations.

The power of ENU (and ethyl methanesulfonate) as a tool for the high-throughput functional annotation of gene sequences has been applied to two primary mutagenesis strategies in mice using either whole mice or mouse embryonic stem (ES) cells: the phenotype-driven mutagenesis screen [5-22] and, more recently, the gene-driven mutagenesis screen [22-30]. An advantage of the phenotype-driven approach is that it does not presuppose the functional roles of any of the genes in the genome, permitting the investigator to identify genes in specific biological processes, pathways, or responses based on the phenotype screens being employed. The strategy yields definitive mutant phenotypes, which cannot always be predicted in gene-driven approaches. Furthermore, phenotype screens have often identified mutations in novel genes, or in known genes for which the resulting mutant phenotypes were not readily predictable from the biochemical functions of the gene products. However, after identifying mice with the desired mutant phenotype, the underlying genetic mutation must still be mapped and cloned. Additionally, phenotype-driven mutagenesis is not a viable approach for recovering mutations in a pre-selected set of genes for which no functional information is available.

The completion of the mouse genome sequence and the development of new efficient methods for the rapid detection of single-nucleotide polymorphisms (SNPs) have made it practical to functionally annotate mammalian genes using high-throughput, cost-effective, gene-driven mutagenesis strategies in the mouse. The benefit of a gene-driven mutagenesis approach is that one can go directly from the DNA sequence information for any gene to the isolation of mutations. Gene-driven mutagenesis screens have been performed both in mouse ES cells [23,26] and in the whole mouse [25,27,29,30]. In practice, the DNAs from large numbers of mutagenized ES-cell clones or the G_1 _progeny of mutagenized male mice are screened for mutations in pre-selected target genes by high-throughput SNP detection methods, and the cryopreserved ES cells or sperm are used to recover mutant mice for phenotype analysis.

These recent developments in ES cells and in mice demonstrate that it is now possible to take full advantage of the availability of the mouse genome sequence and the mutagenicity of ENU to rapidly produce allelic series of mutations in target genes for functional genomics studies. To complement existing embryonic stem-cell-based gene-driven mutagenesis resources, such as gene-trap libraries [31] and ENU-mutagenized CT129/Sv ES cells [23], as well as ENU-mutagenized mice on a mixed genetic background [27,30] or a C3HeB/FeJ inbred genetic background [29], we generated a cryopreserved bank in the C57BL/6J inbred genetic background consisting of DNA, eight pooled organs (for RNA and protein), and sperm from 4,000 G_1 _mice that each carry a load of paternally induced ENU mutations. This Cryopreserved Mutant Mouse Bank (CMMB) is a source of induced, heritable SNPs in virtually every gene in the genome. We used high-throughput Temperature Gradient Capillary Electrophoresis (TGCE) [32] to screen a total of 32 Mbp of PCR-amplified DNA and cDNA from these CMMB mice, which resulted in the identification of 22 ENU-induced mutations in 10 target genes. Because of the advantage of keeping the CMMB on the sequenced C57BL/6J background for future widespread ease of use of the mutant mice, and the inherent difficulties in recovering C57BL/6J mice from cryopreserved sperm by *in vitro *fertilization (IVF), we applied intracytoplasmic sperm injection (ICSI) of cryopreserved sperm for the recovery of mutations.

The unique features of the gene-driven mutagenesis approach presented here include: (1) the CMMB was generated on the defined C57BL/6J genetic background; (2) in addition to genomic DNA, cDNA templates isolated from the pooled, cryopreserved organs of each mouse were also screened for mutations; (3) single-pass, multiplexed TGCE was implemented for high-throughput mutation screening; and (4) live mutant mice were successfully recovered from the cryopreserved sperm by ICSI, an assisted reproduction technique that will significantly extend the life of the sperm for each of the 4,000 males in the CMMB and that will provide for gene-specific point mutations recovered on an inbred C57BL/6J background. Thus, the CMMB will be a useful resource for providing mouse models with a wide range of altered proteins for phenotypic, gene/protein-network, and structural biology-type analyses.

## Results

### Construction of the CMMB

**Figure 1:**
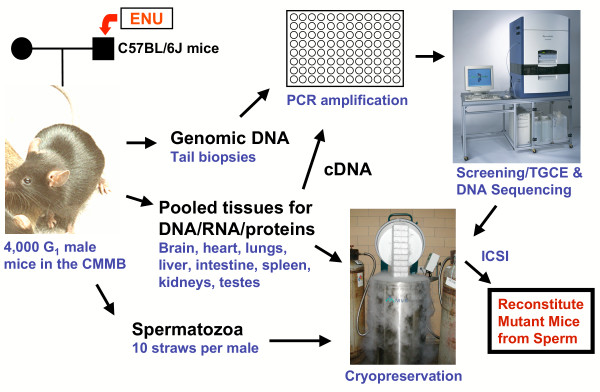
**Gene-driven ENU-induced mutagenesis of any mouse gene using the Cryopreserved Mutant Mouse Bank (CMMB)**. Flowchart showing: generation of the CMMB, PCR amplification of selected genes from the DNA and cDNA templates, mutation screening of PCR products by TGCE and DNA sequencing, and recovery of mutant mice from cryopreserved sperm by ICSI (see text for details).

The CMMB consists of genomic DNA, cDNA, tissue powders, and sperm harvested from 4,000 G_1 _male mice born from the mating of ENU-treated C57BL/6J males to untreated C57BL/6J females. At the time of euthanasia, genomic DNA was prepared from the tail of all 4,000 mice and the sperm was cryopreserved. Additionally, eight organs (brain, heart, spleen, lungs, kidneys, testes, and portions of the liver and small intestine) were harvested, snap frozen in liquid nitrogen, pooled, ground into a powder, and stored in liquid nitrogen. To date, one-fourth of the pooled tissue powder from each of the first 736 mice (~18% of the CMMB) was used to prepare RNA and first-strand cDNA for mutation screening. The 4,000 DNAs and 736 cDNAs were arrayed into 96-well plates for PCR amplification of selected genes and mutation screening by TGCE (Fig. 1).

### Screening the CMMB for ENU-induced point mutations by TGCE

Screening of the CMMB for gene-specific point mutations followed the protocol outlined in Figure 1. Specific segments of selected genes were amplified by PCR from the genomic DNA or cDNA templates in the CMMB. The PCR products were denatured and reannealed to generate heteroduplexes in those samples where an ENU-induced point mutation was present. High-throughput TGCE analysis was then employed to screen the PCR products for the presence of heteroduplexes. TGCE analysis was performed in a 96-capillary array instrument (SpectruMedix SCE9612), in which PCR-amplified DNA and cDNA samples were separated by capillary electrophoresis in a single broad temperature gradient. Samples containing mutations were identified on the basis of altered electrophoretic patterns of homoduplexes and heteroduplexes caused by their different melting equilibria and electrophoretic mobilities [32]. Samples containing heteroduplexes were confirmed by repeating the TGCE analysis only on those samples, and then sequenced to identify the specific base pair mutation.

Prior to the large-scale PCR and TGCE screening of the CMMB for mutations in selected gene fragments (amplicons), the following PCR, TGCE, and DNA sequencing quality-control measures were implemented. PCR primers were designed to amplify genomic DNA containing one or two larger exons and the adjacent splice sites, or to amplify cDNA spanning multiple smaller exons. The amplicons were designed to be ~150–600 bp in length. The optimal annealing temperature of the primers in each PCR or RT-PCR reaction was determined using a gradient thermal cycler, and the products were examined by electrophoresis through agarose gels and by TGCE to evaluate their suitability for mutation screening. The PCR amplicons were also purified and sequenced on both strands with the same primers used to generate the PCR product, in order to confirm the specificity of the PCR reactions.

High-throughput mutation screening of the CMMB was performed by PCR amplification of the DNA and cDNA samples in 96-well plates, followed by the direct analysis of these PCR products for mutations (heteroduplexes) by TGCE. Amplicons were initially analyzed by TGCE in two overlapping temperature gradients (50–58°C and 55–63°C). Subsequent improvements in the gel matrix used for TGCE in the SCE9612 instrument permitted the analysis of all amplicons in a single 50–60°C temperature gradient. Sixteen of the 22 mutations reported here were identified using this new high-viscosity high-resolution gel matrix. Our maximum throughput for TGCE analysis using the new matrix in the SCE9612 was nine 96-well plates per day. The 4,000 DNA samples are in forty-four 96-well plates (92 CMMB samples and 4 control samples per plate). Thus, one amplicon can be screened in the entire CMMB in one week.

**Figure 2:**
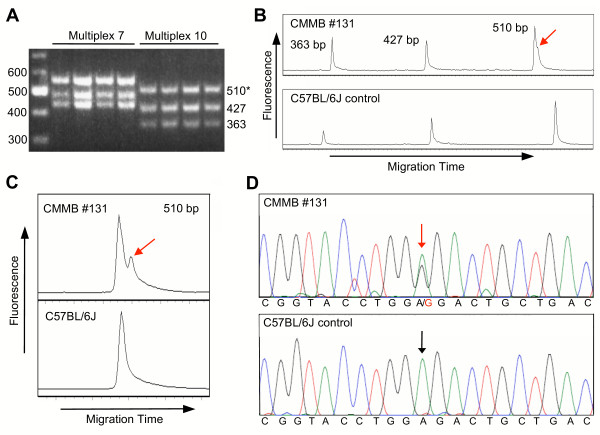
**Identification of a mutation in the CMMB by multiplexed RT-PCR and TGCE, followed by DNA sequencing to identify the specific base pair substitution**. **(A) **Agarose gel electrophoresis of multiplexed RT-PCR reactions. Shown are four representative samples from each of two independent three-fold multiplexed PCR reactions (multiplex 7 and 10) of cDNA templates in the CMMB. Sizes (bp) of molecular weight markers are shown on the left. Sizes (bp) of RT-PCR products of the multiplex 10 reaction are shown on the right. The asterisk indicates the product (510 bp) in which a mutation was identified by TGCE in panel B. **(B) **TGCE electropherogram profiles of three-fold multiplexed PCR products (multiplex 10) derived from CMMB mouse #131 (top) and C57BL/6J control (bottom) cDNA templates. A mutation (heteroduplex, red arrow) was identified in CMMB #131 in a 510-bp product derived from the *Ap2a1 *gene. **(C) **The mutation was confirmed by repeating the RT-PCR and TGCE analysis of only the 510-bp product amplified from the CMMB #131 cDNA sample. **(D)** DNA sequence analysis of the 510-bp products amplified from the CMMB #131 and control cDNAs revealed an A-to-G nucleotide substitution (red arrow) in the *Ap2a1 *gene in the CMMB #131 sample, which causes a Glu414Gly amino acid substitution in the encoded protein.

We increased the throughput of mutation detection an additional three-fold by routinely performing PCR amplification and TGCE analysis of multiple amplicons simultaneously (i.e., multiplexing). A typical three-fold multiplex experiment with cDNA templates is shown in Figure 2. Three pairs of PCR primers were combined in a single PCR reaction for each of the CMMB cDNA templates (Fig. 2A). The PCR products were then directly analyzed for the presence of heteroduplexes by TGCE, which resulted in the identification of a mutation in one of the three multiplexed amplicons in CMMB sample #131 (Fig. 2B). This mutation was confirmed by repeating the PCR and TGCE analysis of sample #131 (Fig. 2C), and direct DNA sequence analysis of the PCR product identified an ENU-induced A-to-G mutation in the heterozygous DNA sample (Fig. 2D). We also determined that the throughput for mutation detection can be increased an additional 10-fold by pooling the DNA or cDNA templates (placing templates from 10 mice in each well) prior to PCR and TGCE analysis [[32]; and data not shown].

**Figure 3:**
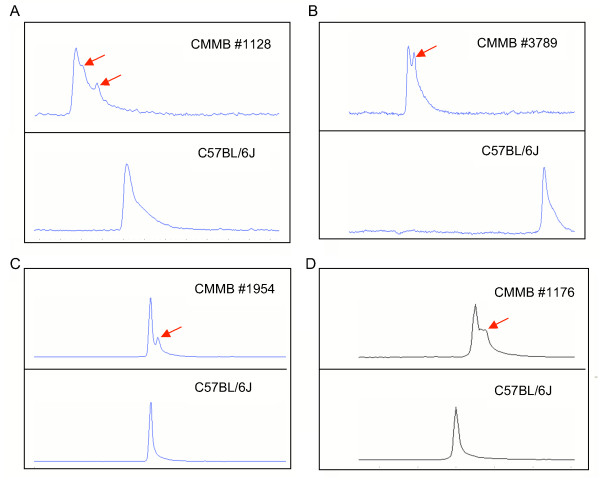
**Four categories of electropherogram profiles generated by high-throughput TGCE mutation screening of DNA and cDNA samples in the CMMB**. The 22 ENU-induced mutations identified in this study (see Table 1) by TGCE analysis of heteroduplexed PCR products produced electropherogram patterns that can be divided into four general categories based on the number and shape of the peaks. Shown are representative electropherograms from each category, which include: **(A) **three peaks (CMMB #1128, 484-bp amplicon, *Scnm1*); **(B) **two peaks of approximately equal intensity (CMMB #3789, 484-bp amplicon, *Scnm1*); **(C) **two peaks with lower intensity of the heteroduplex peak (CMMB #1954, 336-bp amplicon, *Usp29*): and **(D) **a shoulder on the main peak (CMMB #1176, 335-bp amplicon, *Antxr1*). In each of the four panels the electropherogram on the top is from the indicated CMMB sample with a heterozygous ENU mutation and the electropherogram on the bottom is from an untreated C57BL/6J control sample. All patterns shown were obtained from first-pass TGCE screening of unpurified PCR products using a single 50–60°C gradient. Heteroduplex molecules (arrows) denature faster than the homoduplexes as temperature increases during electrophoresis. The distribution of electropherogram patterns observed for the 22 mutations can be categorized as follows: **(A) **2, **(B) **4, **(C) **12, and **(D) **4 mutations. Thus, 82% of the profiles exhibited two or more peaks and the remaining 18% were shoulders.

Mutation screening by TGCE with a single broad range of temperatures eliminates the need to determine the optimum melting temperature for any amplicon, and also increases the efficiency and throughput of the screen. As a result of this broad temperature range, the four peaks that are expected in an electropherogram following TGCE analysis of a DNA sample with a heterozygous point mutation (both heteroduplexes, and wild-type and mutant homoduplexes) are, in practice, typically compressed into two peaks. Of the 22 mutations reported here (Table 1), two were detected as three peaks, 16 were detected as two peaks, and the remaining four mutations were seen as "shoulders" on the primary peak, all following first-pass TGCE analysis (Fig. 3).

**Table 1:** Mutations identified in the CMMB. Types of ENU-induced gene mutations identified by TGCE screening of DNA and cDNA templates from 4,000 C57BL/6J mice in the CMMB, and recovery of mutant lines from the cryopreserved sperm by ICSI.

Gene symbol^a^	Amplicon size (bp)	Template (D-DNA C-cDNA)	Number of individuals screened^b^	Mbp screened	Mutations identified, (mouse #)^c^	GenBank accession number	Base mutation^d^	Amino acid change	Type of mutation	Rederived by ICSI
***Mc1r***	579	D	1585*	0.917	0	NM_008559				
***Mc1r***	446	D	1667*	0.743	1 (1110)	NM_008559	T781C	Phe256Ser	Nonconservative	Yes
***Mc1r***	527	D	1671*	0.880	1 (997)	AF176016	A115G	Promoter	Promoter^e^	Yes
***Zfp111***	352	D	1686*	0.593	0	NM_019940				
***Zfp111***	327	D	1250*	0.409	1 (803)	NM_019940	G2182A	3' UTR	3' UTR^f^	Yes
***Scnm1***	394	D	3641	1.434	0	NM_027013				
***Scnm1***	484	D	3400	1.645	1 (1128) 1 (3789)	NM_027013 AC140190	T428C T19199C	Ile112Thr Intronic	Nonconservative Intron 5^g^	Yes
***Scnm1***	372	D	3800	1.414	1 (2364)	NM_027013	A594G	Ser167Ser	Silent	
***Usp29***	300	D	3884	1.165	1 (3909)	NM_021323	G1850A	Glu124Lys	Nonconservative	Yes
***Usp29***	336	D	3544	1.191	1 (1954)	NM_021323	T2933A	Cys485Ser	Conservative	
***Usp29***	298	D	3621	1.079	1 (573)	NM_021323	A3516G	Lys679Arg	Conservative	
***Zim1***	298	D	2037*	0.607	1 (195)	NM_011769	A808G	Lys176Arg	Conservative	
***Zim1***	298	D	3720	1.109	1 (2277)	NM_011769	A1017G	Lys246Glu	Nonconservative	
***Zim1***	302	D	3726	1.125	1 (1027) 1 (3119)	NM_011769 NM 011769	T1660A A1610G	Val460Glu Gly443Gly	Nonconservative Silent	
***Zim1***	302	D	3801	1.148	0	NM_011769				
***Myd88***	429	D	3725	1.598	0	NM_010851				
***Myd88***	196	D	3669	0.719	0	NM_010851				
***Myd88***	497	D	3739	1.858	1 (364)	NM_010851	G604A	Val175Met	Conservative	
***Myd88***	245	D	3836	0.940	1 (1569)	NM_010851	T899A	Ile273Lys	Nonconservative	Yes
***Ap2s1***	424	D	3754	1.592	0	NM_198613				
***Capsl***	372	D	3694	1.374	1 (661)	NT_039618	T418425A	Intronic	Intron 2^h^	
***Capsl***	344	D	2422*	0.833	1 (1813)	NM_029341	G665A	Leu125Leu	Silent	
***Antxr1***	419	D	3726	1.560	1 (832)	AC153853	A188590G	Intronic	Intron 1^i^	
***Antxr1***	335	D	3739	1.253	1 (1176)	AC153853	T94846A	Intronic	Intron 4^j^	
***Antxr1***	157	D	3841	0.603	0	NM_054041				
***Antxr1***	363	C	714*	0.259	0	NM_054041				
***Antxr1***	490	C	728*	0.357	0	NM_054041				
***Antxr1***	489	C	718*	0.351	0	NM_054041				
***Ap2a1***	510	C	713*	0.364	1 (131)	NM_007458	A1446G	Glu414Gly	Nonconservative	Yes
***Ap2a1***	543	C	732*	0.397	1 (663)	NM_007458	T796A	Asn197Lys	Nonconservative	Yes
***Ap2a1***	442	C	729*	0.322	0	NM_007458				
***Ap2a1***	531	C	730*	0.388	0	NM_007458				
***Ap2a1***	351	C	707*	0.248	0	NM_007458				
***Pak4***	503	C	441*	0.222	1 (61)	NM_027470	C1094T	Pro283Ser	Nonconservative	Yes
***Pak4***	477	C	728*	0.347	0	NM_027470				
***Pak4***	459	C	707*	0.325	0	NM_027470				
***Pak4***	412	C	728*	0.300	0	NM_027470				
***Pak4***	447	C	731*	0.327	0	NM_027470				
TOTAL				31.996	22					

^a^*Mc1r*, melanocortin 1 receptor; *Zfp111*, zinc finger protein 111; *Scnm1*, sodium channel modifier 1; *Usp29*, ubiquitin specific protease 29; *Zim 1*, zinc finger, imprinted 1; *Myd88*, myeloid differentiation primary response gene 88; *Ap2s1*, adaptor-related protein complex 2, sigma 1 subunit; *Capsl*, calcyphosine-like; *Antxr1*, anthrax toxin receptor 1; *Ap2a1*, adaptor protein complex AP-2, alpha 1 subunit; *Pak4*, p21 (CDKN1A)-activated kinase 4

^b^Asterisk indicates that only a portion of the CMMB was screened for the amplicon

^c^Number in parentheses indicates the CMMB animal number in which the mutation was identified

^d^Bases are numbered according to corresponding GenBank accession number and refer to the sense strand

^e^-681 bp upstream of start codon

^f^+7 bp downstream of stop codon

^g^intron 5, +62 bp downstream of end of exon 5

^h^intron 2, -7 bp upstream of start of exon 3

^i^intron 1, -33 bp upstream of start of exon 2

^j^intron 4, +75 bp downstream of end of exon 4

### Types of mutations and mutation frequency in the CMMB

Genomic DNA and cDNA templates were used to screen for mutations in 38 amplicons from 11 genes by TGCE analysis (Table 1). The entire CMMB (DNA) was screened for 18 of these amplicons, whereas a portion of the bank (DNA or cDNA) was screened for the remaining 20 amplicons, resulting in a total 32.0-Mbp screen. Amplicon sizes ranged from 157 to 579 bp. A total of 22 point mutations was identified in 10 of the 11 genes, which equals a mutation frequency of 1 mutation for every 1.454 Mbp screened. Thus, on average, screening a 364-bp amplicon in all 4,000 samples in the CMMB will yield one new mutation. The number of mutations identified per amplicon ranged from none to two. Of the 22 mutations identified: (a) 19 were identified in genomic DNA templates and the remaining three in cDNA templates; (b) 17 occurred in A-T base pairs and five in G-C base pairs; (c) 16 were transitions and six were transversions; and (d) 16 occurred in the coding regions of genes, one was in the promoter, one was in the 3' UTR, and four were in introns. Of the 16 mutations that occurred in the coding regions of genes, 13 were missense mutations (nine nonconservative and four conservative amino acid substitutions) and three were silent mutations.

Two sequence homology-based tools, Sorting Intolerant From Tolerant (SIFT) [33] and Polymorphism Phenotyping (PolyPhen) [34], were used to predict the potential impact of the 13 nonsynonymous DNA changes on protein structure and function (Table 2). Generally speaking, these programs predict whether an amino acid substitution will impact protein function based on sequence comparisons among evolutionarily related proteins. The potential impact of each nonsynonymous change is reported as tolerated or not tolerated (SIFT), or benign, possibly damaging, or probably damaging (PolyPhen).

**Table 2:** SIFT and PolyPhen predictions of the possible impact of 13 amino acid variants in the CMMB on protein structure and function.

Gene (protein ID)	Amino acid change	SIFT score^a^	SIFT prediction	PolyPhen prediction
***Mc1r ***(NP_032585)	Phe256Ser	(0.00)	(Not Tolerated)	Possibly Damaging
***Scnm1 ***(NP_081289)	Ile112Thr	(0.44)	(Tolerated)	Benign
***Usp29 ***(NP_067298)	Glu124Lys	(0.05)	(Tolerated)	Benign
***Usp29 ***(NP_067298)	Cys485Ser	0.01	Not Tolerated	Probably Damaging
***Usp29 ***(NP_067298)	Lys679Arg	0.05	Tolerated	Benign
***Zim1 ***(NP_035899)	Lys176Arg	0.62	Tolerated	Benign
***Zim1 ***(NP_035899)	Lys246Glu	(0.30)	(Tolerated)	Benign
***Zim1 ***(NP_035899)	Val460Glu	0.18	Tolerated	Benign
***Myd88 ***(NP_034981)	Val175Met	0.01	Not Tolerated	Benign
***Myd88 ***(NP_034981)	Ile273Lys	0.29	Tolerated	Probably Damaging
***Ap2a1 ***(NP_031484)	Glu414Gly	0.23	Tolerated	Possibly Damaging
***Ap2a1 ***(NP_031484)	Asn197Lys	0.36	Tolerated	Possibly Damaging
***Pak4 ***(NP_081746)	Pro283Ser	0.42	Tolerated	Possibly Damaging

^a^SIFT scores indicate if amino acid changes are predicted to be Not Tolerated (<0.05) or Tolerated (≥0.05). SIFT scores and predictions in parentheses should be interpreted with caution because the median sequence conservation score was >3.25.

### Recovery of mutant mice from cryopreserved sperm by ICSI

The CMMB includes 10 straws of sperm from each mouse. Upon thawing one straw for ICSI, the sperm was washed with NIM medium (see Methods) to remove the cryoprotectant, resuspended in NIM medium, aliquoted into 20 cryovials, refrozen, and stored in liquid nitrogen. One of these 20 smaller aliquots of sperm was used per morning of ICSI, thus providing for ~200 days of ICSI for each G_1 _male in the CMMB.

As an additional quality-control measure prior to the recovery of mutant mice with the cryopreserved sperm, the mutation of interest was re-sequenced in the sperm DNA. ICSI was performed essentially as described [35] with minor modifications (see Methods). To date, live mutant mice have been recovered for nine of the 22 mutations by injecting the cryopreserved C57BL/6J sperm into B6D2F1 ova (Table 1). Most of our injections have been performed with B6D2F1 ova because of the increased efficiency of recovering live mice from cryopreserved C57BL/6J sperm using hybrid ova [36]. However, ICSI was also performed with C57BL/6J ova for one of these nine mutations and live mice were recovered, demonstrating the ability to recover CMMB mice on the C57BL/6J genetic background. Future efforts will be directed toward recovering mutant CMMB mice with only C57BL/6J ova.

### Genotyping and breeding mutant mice for phenotype analysis

G_2 _mice recovered by ICSI with C57BL/6J G_1 _sperm and B6D2F1 ova were genotyped for the presence of the heterozygous point mutation with the same PCR and TGCE methods used to identify the mutation originally in the CMMB. Heterozygous G_2 _mutant mice were then mated to wild-type C57BL/6J mice to produce heterozygous G_3 _offspring. Multiple pairs of heterozygous G_3 _mice were intercrossed to produce 20 G_4 _mice for phenotype analysis. Offspring from the G_3 _intercrosses were genotyped by TGCE or DNA sequencing of PCR products (see Methods).

The nine lines of mutant mice recovered by ICSI are at various stages in this process, ranging from heterozygous G_2 _mice, to homozygous mutant G_4 _animals that are undergoing phenotype analysis. The results of the phenotype analyses will be described elsewhere. We are also in the process of cryopreserving embryos for the lines of mutant mice recovered by ICSI. The frozen embryos will then be advertised on our web site at ORNL [37], which can also be searched via the International Mouse Strain Resource web site [38].

## Discussion

In this study we generated a bank of DNA, tissues, and sperm from 4,000 male progeny of ENU-mutagenized C57BL/6J mice, employed TGCE as a high-throughput and cost-effective gene-driven approach for identifying mutations, and used ICSI to recover live mice from cryopreserved sperm of males carrying specific mutations. An initial 32.0-Mbp screen of genomic DNA and cDNA templates in the CMMB resulted in the identification of 22 point mutations, with a per-base-pair mutation frequency of 1 in 1.454 Mbp (Table 1). This mutation frequency is comparable to the frequency reported for other gene-driven ENU mutagenesis screens in mice [27,29,30]. Of these 22 mutations, 13 of them (59%) were missense mutations (nine nonconservative and four conservative) with the potential to cause functional changes in the encoded proteins. Therefore, one missense mutation was identified in the CMMB per 2.46 Mbp screened, or one potential functional change for every 615 bp screened in all 4,000 mice. If we impose a stricter definition and consider that only the nonconservative amino acid substitutions are likely to cause functional protein changes, then nine of the 22 mutations (41%) reported here are in this category. Using this stricter definition, one functional change was identified in the CMMB for each 3.56 Mbp screened or, on average, one functional change (nonconservative amino acid substitution) was identified for every 890 bp screened in all 4,000 mice. Based on these data and the current size of the Oak Ridge CMMB, screening an entire gene with an open reading frame of ≥ 2.7 kb would result in ≥ 3 functional alleles. To identify functional allelic series' in smaller genes, or in a small discrete region of a gene, it would be advantageous to proportionately expand the size of the CMMB (e.g., a bank of 10,000 mice would yield one functional change for any 356-bp amplicon).

The SIFT and PolyPhen programs predicted that seven of the 13 nonsynonymous DNA changes might have an effect on protein structure and function. The predictions made by these tools were often in disagreement and should therefore be viewed with caution. However, these types of predictions do offer additional information when trying to prioritize the order in which lines of mutant mice will be recovered from frozen sperm for phenotype analysis.

### TGCE as a high-throughput screening method

TGCE [32] was employed as a rapid, efficient, and cost-effective method for identifying ENU-induced point mutations in the CMMB. The relatively new TGCE technology has been used for clinical applications to identify mutations in Factor V [39], hepatitis C virus [40], mtDNA in nonmelanoma skin cancer samples [41], and the cyctic fibrosis transmembrane conductance regulator gene [42]. More recently, as part of a phenotype-driven ENU mutagenesis screen, our group demonstrated that TGCE is an effective method for identifying mutations in candidate genes associated with mutant mouse phenotypes that map to defined chromosome intervals [21], and two other groups have applied TGCE to gene-driven ENU mutagenesis screens [29,30].

In the present study, we also demonstrated that TGCE is a powerful tool for identifying rare mutations in pre-selected genes within a large bank of mutagenized DNA samples. There are numerous advantages to using TGCE for gene-driven ENU-induced mutagenesis screens. TGCE analysis has a greater than 95% mutation-detection rate [39,41,42]. TGCE permits direct analysis of very small volumes of unpurified PCR products that can also be multiplexed, even amplicons with different melting temperatures. Mutations are identified from first-pass screening without the need to predetermine the melting temperatures of different amplicons. Finally, TGCE is sensitive enough to permit sample pooling. In our hands using the SCE9612, we were able to analyze nine 96-well plates per day. We demonstrated a routine ability to perform three-fold multiplexing of samples, and we determined that we could identify single ENU mutations from pools of 10 DNA samples. Experiments are under way to combine PCR/TGCE multiplexing and sample pooling to significantly increase throughput. For example, by pooling eight samples (the columns in each 96-well plate), the 4,000 CMMB samples could be reduced to six 96-well plates. If three amplicons (300, 400, and 500 bp) were screened by PCR and TGCE simultaneously (three-fold multiplex, see Fig. 2) in these pools of eight DNA samples, a 4.8-Mbp screen could be completed in a single day. At our current mutation frequency of 1 in 1.454 Mbp in the CMMB, this could result in the identification of three ENU-induced mutations per day, with at least one of them causing a nonconservative amino acid change in the encoded protein.

### Screening both genomic DNA and cDNA templates

In addition to the 4,000 tail-DNA samples in the CMMB, we began preparing and screening cDNA samples from the corresponding 4,000 tissue powders. There are advantages to having both DNA and cDNA for mutation screening. The DNA templates are more abundant and less expensive to produce than cDNA templates, and they permit the screening of exons and their splice junctions. Since it is desirable in terms of throughput to screen large amplicons that contain mostly coding sequences, DNA templates are best suited for screening large single exons, or two to three small exons separated by small introns. However, because the average exon size in mouse is only ~250 bp (National Center for Biotechnology Information), using cDNA templates allows one to design large amplicons that consist only of coding sequences. Mutation screening of cDNAs permits rapid coverage of the gene's entire coding region, which is especially useful if one wants to screen a conserved functional motif(s) that spans numerous small exons spread across a large genomic distance. One potential drawback to the use of cDNA templates is that nonsense-mediated mRNA decay could potentially decrease the efficiency of identifying ENU-induced mutations that introduce premature termination codons, although this type of mutation will occur infrequently compared to those that cause amino acid substitutions. The possible decreased efficiency of identifying such truncation mutations in our screen, which may result in null alleles, would be minimized if a knockout mutation already existed for the gene of interest.

### Rationale for overcoming inbred C57BL/6J fertility problems by ICSI

The CMMB was deliberately generated with inbred C57BL/6J mice so it would be in the "reference" genetic background for the mouse genome. Although recovering live mice from cryopreserved C57BL/6J sperm by IVF is very inefficient compared to some other mouse strains [43], this problem is overcome with ICSI [36,44,45]. We used ICSI successfully to recover live mice from the cryopreserved CMMB sperm, including frozen sperm that was thawed and refrozen without cryoprotectant, using both B6D2F1 ova and C57BL/6J ova. Some lines were recovered with only one or two sessions of ICSI injections. Other lines required many attempts, but we have not yet failed to recover any line. Although ICSI requires a higher level of technical expertise and more hands-on time compared to IVF, it is useful for recovering live mice from highly mutagenized, frozen, C57BL/6J sperm, and it significantly extends the life of the sperm bank. This latter point is not a trivial issue, given that each of the 4,000 sperm samples in the bank came from a single G_1 _male. Using ICSI to recover live mice from frozen sperm in the CMMB, we effectively have enough sperm from one CMMB male to perform a minimum of 200 days of ICSI. More importantly, the extra effort involved in mutation recovery with ICSI will be well worth it for the long-term usefulness of the C57BL/6J mutant mice, especially since most other mutations in the mouse genetic resource are also on this background. Thus, in future tests of phenotypic effects of different CMMB alleles within an allelic series, or when multiple mutations from the CMMB are combined with each other or with mutations from different sources into one line of mice to examine genetic interactions, we expect background effects to be minimal.

### "Off-target" mutations

An important consideration in any type of random mutagenesis screen is the impact that mutation load and "off-target" mutations will have on the ability to determine that a specific mutation of interest is solely responsible for the observed mutant phenotype. For this reason, we elected to treat C57BL/6J mice with a fractionated dose of ENU (3 × 85 mg/kg) that is less than the dose that can be tolerated by this strain of mice [46]. The objective was to obtain a mutation load that was high enough to make screening efficient, but not so high that off-target mutations became a significant concern. Assuming that all missense mutations we identified have the potential to produce a functional protein change, our estimate of functional mutation frequency in the Oak Ridge CMMB is 1 in 2.46 Mbp. Also assuming a 1,600-centimorgan 2.7-Gigabase-pair haploid mouse genome of which 3% is coding, each G_1 _male will have approximately 33 functional heterozygous mutations, with an average genetic distance between mutations of 48 centimorgans, or an average of 2 mutations per autosome. Therefore, we can assume that nearly all of these mutations will segregate independently, and the likelihood of having tightly linked, off-target *functional *mutations that confound phenotype interpretation will be rare. Even so, it is expected that G_2 _mice will inherit half of the mutations (17) from the G_1 _sperm, and that G_3 _mice will inherit half of these (9) from the G_2 _mice upon backcross to untreated C57BL/6J mice. Only 25% of these 9 mutations (2) are expected to be present in both mice of a G_3 _intercross breeding pair. Therefore, when G_3 _mice are intercrossed to produce 20 G_4 _mice for phenotype analysis, it is possible that 1 or 2 off-target mutations could become homozygous in a portion of these mice. However, a false association of the mutant phenotype with the target gene due to the co-segregation of an off-target mutation is extremely unlikely. Such a false association would require that homozygosity for the off-target mutation be completely concordant with homozygosity for the target mutation. Additionally, phenotype-genotype associations will be confirmed in offspring from multiple pairs of G_3 _mice, effectively eliminating the chance of an occurrence of co-homozygosity for an off-target mutation in all G_4 _target-mutation homozygotes. Finally, and importantly, effects of off-target mutations can be ruled out by performing phenotype analysis in compound heterozygous mice in which the TGCE-detected ENU allele is in *trans *with a null mutation generated by homologous recombination in ES cells.

## Conclusions

The gene-driven mutagenesis approach presented here offers significant advantages in performing time- and cost-effective mutagenesis in mice, in screening for mutations in target genes, and in re-deriving mutant mice. Only a single generation of mice was needed to make the CMMB, large numbers of G_1 _mice were generated and euthanized in a short time, and the cost of making the bank was a one-time investment. The design of the CMMB ensures that none of the mutations to be recovered from the bank are dominant lethals, since all of the heterozygous mutations were present in the G_1 _individuals used to make the bank. Using the CMMB for mutation screening offers the advantage of screening for mutations in any target gene in the genome (except those on the X chromosome) using both DNA and cDNA templates, and recovering only those lines of mice with pre-selected mutations from the frozen sperm bank. The genes screened here were selected on the basis of their importance in different biological processes, but gene-driven ENU-based mutagenesis will be especially useful for systems biology-based interrogations of distinct genetic pathways and networks. The primary advantages of TGCE for mutation screening over other SNP detection methods, such as denaturing high-performance liquid chromatography, lie in its ability to detect mutations in unpurified, multiplexed PCR amplicons with different denaturation temperatures, without the need to predetermine the denaturation temperature for any amplicon. Performing ICSI with frozen sperm from the G_1 _mice in the CMMB provides numerous opportunities to recover live mutant mice from multiple different mutations that may occur in the same cryopreserved sperm sample, and also allowed us to make the entire bank on the widely used C57BL/6J reference genetic background. In the future, mutations in different genes may be crossed into one line of mice to better define protein-protein interactions in protein complexes, to observe effects of mutations in certain "sensitized" backgrounds, and to model multi-gene disease syndromes in humans without the effects of modifying genes introduced by different genetic backgrounds. Thus, the generation of allelic series' of point mutations by gene-driven ENU-induced mutagenesis in mice provides an important complement to knockout and conditional alleles in our quest to determine the whole-animal biological functions of all mammalian genes.

## Methods

### ENU mutagenesis

Seven groups of C57BL/6JRn male mice ≥ eight weeks old (a total of 425 males) were each given three weekly intraperitoneal (IP) injections of 85 mg/kg ENU (Sigma-Aldrich, St. Louis, MO) for a total fractionated dose of 255 mg/kg, as described [5]. Males were routinely bred to untreated C57BL/6JRn females. Of the 425 injected males, 319 males each produced an average of 13 G_1 _males (range of 1 to 43), for a total of 4,000 G_1 _males that comprise the CMMB. C57BL/6JRn mice are C57BL/6J mice purchased from The Jackson Laboratory in 1986 and maintained continuously at the Oak Ridge National Laboratory as an inbred stock; Rn = Rinchik. All use of mice in this study was conducted under approved Institutional Animal Care and Use Committee protocols and in accordance with the *Guide for the Care and Use of Laboratory Animals *(National Research Council).

### Genomic DNA preparation

Following euthanasia, duplicate samples of genomic DNA were prepared from large segments of the tail from each of the 4,000 mice. For each DNA sample, approximately 4 cm of the tail, cut into two pieces, was placed into a Serum Separation Tube (Becton Dickinson Vacutainer SST tube, 13 × 100 mm, Fisher, Pittsburgh, PA). Each tail sample was covered with 1.5 ml of tail lysis buffer (10 mM Tris-HCl, pH 7.6, 10 mM EDTA, 100 mM NaCl, 0.5% SDS, 0.1 mg/ml Proteinase K), covered with parafilm and incubated overnight at 50°C. After incubation, 1.5 ml of buffered phenol (500 g of JT Baker crystal phenol, 100 ml of 2M Tris pH 8.0, 130 ml of double-distilled water, 25 ml of m-cresol, 1 ml of 2-mercaptoethanol and 0.5 g of 8-hydroxyquinoline) were added to each SST tube and the samples were mixed thoroughly in a rack vortexer. Tubes were placed in a Beckman Model TJ-6 tabletop centrifuge and spun at full speed (2800–3000 rpm) for 20 minutes. Following centrifugation, the DNA was extracted twice with chloroform; 1.5 ml of chloroform were added to each tube, vortexed, centrifuged at full speed for 20 minutes, and repeated. The DNA solution on top of the gel barrier was transferred to a 13-ml Sarstedt tube (Sarstedt, Newton, NC), and DNA was precipitated by addition of two volumes of 95% EtOH. The precipitated DNA was transferred to a 1.5 ml microcentrifuge tube with a sterile pipette tip, air dried for 10 min to evaporate the ethanol, resuspended in 400 μl of TE (10 mM Tris, 1 mM EDTA, pH 8.0), and stored at 4°C. In preparation for PCR and TGCE screening, the concentrations of the DNA samples were determined with a spectrophotometer. On average, each sample yielded a total of approximately 500 μg of genomic DNA.

### Tissue cryopreservation

The brain, heart, spleen, lungs, kidneys, testes, and portions of the liver and small intestine were harvested from each of the 4,000 G_1 _mice, snap frozen in liquid nitrogen, pooled together into a 50 ml polypropylene tube (BD Falcon BlueMax) on dry ice, and ground into a coarse powder in liquid nitrogen with a mortar and pestle. The tissue powder from each mouse was distributed into four 2-ml Nunc cryotubes on dry ice and then stored in two liquid-nitrogen freezers.

### Sperm cryopreservation

Sperm cryoprotectant consisted of 18.0% D-(+)-Raffinose pentahydrate (Sigma-Aldrich R7630) and 3.0% Dehydrated Skim Milk (Difco/BD 0032-17-3) in sterile water (Sigma-Aldrich W1503). Water was warmed to approximately 40°C and raffinose was added with stirring until completely dissolved. Skim Milk was added with continued stirring until dissolved. The solution was centrifuged at 10,000 × g, 20°C, for 20 min. The supernatant was filtered through a 0.45-micron filter, aliquoted, and stored at -20°C. Individual aliquots were warmed on the day of use to ensure that the raffinose and skim milk were in solution.

For each of the 4,000 G_1 _males (≥ 12 weeks old), the epididymides and vas deferentia were removed and placed in 1 ml of sperm cryoprotectant in a small petri dish at 37°C. Sperm was stripped from the vas deferentia into the cryoprotectant by holding one end with a pair of forceps and gently running another pair of forceps along the length of the vas. The empty vas deferentia were discarded. The epididymides were minced in the cryoprotectant with two pair of forceps. After the dish remained undisturbed at 37°C for 2–3 minutes to allow the sperm to "swim out", the pieces of tissue were removed with forceps and 100 μl samples of sperm in cryoprotectant were drawn into 10 cryostraws (0.25 ml capacity, #A201; IMV, L'Aigle, France), which were heat sealed on the ends. The sperm samples were frozen in liquid nitrogen vapor for 10 minutes, followed by plunging the straws into liquid nitrogen in two Dewar flasks for storage.

### Preparation of RNA and first-strand cDNA templates from tissue powders

One of the four tubes of tissue powder (~0.5–1.0 g) from each mouse was used to prepare total RNA using the RNeasy Maxi kit (Qiagen, Valencia, CA). The manufacturer's protocol was used with the following modifications to improve purity and yield of RNA. The tissue powder was homogenized in the lysis buffer for 45–60 sec using a Polytron rotor-stator homogenizer, and 50% ethanol was added to the homogenized lysate rather than 70% ethanol. After the second wash step, the RNeasy columns were centrifuged an additional 5 min with the lids off to remove residual traces of ethanol. The RNA was eluted from the column by adding 0.8 ml of RNase-free water that was preheated to 50°C, incubated for 5 minutes, centrifuged, and then repeated with another 0.8 ml of water. The eluted RNA was divided into two 1.5-ml tubes and stored at -80°C. With this method, 1.5–3.5 mg of total RNA was purified from each tube of tissue powder from the first 736 CMMB mice (~18% of the entire bank).

First-strand cDNA templates were prepared as follows: 6 μg of total RNA, 0.9 μg of Random Hexamers (Amersham Biosciences, Piscataway, NJ) or Random Primers (Invitrogen, Carlsbad, CA), 0.5 μg of Oligo(dT)_12–18 _(Invitrogen), and RNase-free water, in a total volume of 40.5 μl, were incubated at 65°C for 4 min and then immediately placed on ice. A 60 μl cDNA-synthesis reaction was completed by adding 12 μl of 5× First strand buffer [250 mM Tris-HCl (pH 8.3), 375 mM KCl, 15 mM MgCl_2_] (Invitrogen), 3 μl of 0.1 M DTT, and 3 μl of a 40 mM mixture of dNTPs (10 mM of each dNTP), and 1.5 μl (300 U) of Superscript III reverse transcriptase (Invitrogen). The reaction was incubated at 25°C for 10 min, followed by 50°C for 50 min, and finally 70°C for 15 min.

### PCR amplification of DNA and cDNA templates

For each of the 4,000 CMMB DNAs, 1 ml dilutions at 5 ng/μl were prepared. Aliquots of each diluted DNA (2 μl) were transferred into 96-well master plates (AB-0600, ABgene, Rochester, NY) and served as the templates for mutation screening. The DNAs were arrayed into forty-four 96-well plates, with 92 DNAs in each plate except for the last plate, which contained 44 DNAs. The 736 first-strand cDNAs were arrayed into eight 96-well plates (0.8 μl of first-strand cDNA reaction per well), with 92 cDNAs in each plate. Well number 93 (row H, column 9) in the DNA and cDNA plates contained unmutagenized C57BL/6J DNA (negative control). Well number 94 (H10) contained DNA from a C57BL/6J mouse with a known ENU-induced heterozygous point mutation (positive control for PCR and TGCE). Replica plates were made in batches of 10 for every DNA and cDNA plate. The plates were incubated at 70°C for 10–15 min to dry down the samples, sealed with TR100 adhesive seals for microtiter plates (Marsh, Rochester, NY), and stored at -20°C until ready for use in PCR or RT-PCR.

PCR primers (19–24 nt), designed to produce amplicons ~150–600 bp in length, were selected using Mac Vector (Accelrys, San Diego, CA), Primer3 [47], or by visual examination of the DNA or cDNA sequence. Primer sequences were examined by BLAST analysis [48] against the mouse genome in order to select unique primer pairs. The DNA sequences of the primer pairs used for PCR amplification of the amplicons listed in Table 1 are shown in Table 3. The optimal PCR conditions for each pair of primers (or multiplexed pairs of primers) were determined with wild-type C57BL/6J DNA or cDNA in a gradient thermal cycler (Eppendorf Gradient Mastercycler). When screening the cDNA archive by multiplexed PCR, primer dilution was used to generate approximately equal amounts of RT-PCR products from target genes with different levels of expression.

**Table 3:** PCR primer sequences used to amplify DNA (D) or cDNA (C) templates for mutation screening. The order of the amplicons listed is the same as in Table 1.

Gene symbol	Amplicon size (bp)	Template	Forward primer (5' to 3')	Reverse primer (5' to 3')
***Mc1r***	579	D	gaggatccttcctgacaagactatgtcca	aacggctgtgtgcttgtagtagg
***Mc1r***	446	D	tcgtctccagcaccctctttatc	gagtcgacgatcaccaggagcacagcagca
***Mc1r***	527	D	gcacacttctaatggagagtg	ggctcaggtagagacatgcc
***Zfp111***	352	D	ccagagagaaataatgcccc	caagaaccctgggctctcc
***Zfp111***	327	D	ggttcagtcaggtctcacac	caggattgatataatgctcc
***Scnm1***	394	D	aggcacagcgtctcaaagtattgt	atagggtagtaggggtcaccactc
***Scnm1***	484	D	ggcaagaagcatttgtccagtaag	tttctcagaatacaatctggagagc
***Scnm1***	372	D	tgagttcccgtcagccaggacttc	ctcactcaccttcggagggtaagg
***Usp29***	300	D	ccttactcatcgacaagttatc	tggaggaggatggttctgtctt
***Usp29***	336	D	gggtctgctggcaccaaaaggt	ggcaacaagtcagtggtaact
***Usp29***	298	D	gggtcttcaagaggttccagag	gagcctgtaattctgaagatca
***Zim1***	298	D	ggaagaagacaggggataattcc	ggagcgctctgtggtgttgtag
***Zim1***	298	D	atcttcgggtcaaacatcagca	gtagtgtgtgaggaagtatgaga
***Zim1***	302	D	tggagagtgtaacaagtgcttc	ggtgcttgagaagggctactttg
***Zim1***	302	D	cccggagtgtgggaaagtcttc	ggtgaatcagcagggtagccagt
***Myd88***	429	D	ggctggcaggagacttaagg	caggaagcacgtttcctcac
***Myd88***	196	D	cacccttctcttctccacag	gcccacctattctacctagg
***Myd88***	497	D	ccttctgcagaggctgattg	ccaaagcaggcctaagcttac
***Myd88***	245	D	cgtggtcctaataccacacc	ggaggcaagcggaagaacac
***Ap2s1***	424	D	gccttgtctgtacctgtctc	ggacgagcaggcaggttggtc
***Capsl***	372	D	gaggtaaacctagggcttctg	ctgccacgctgtcaataccg
***Capsl***	344	D	gcacatgcatttctccatgg	gctttgtaaggctctgaacc
***Antxr1***	419	D	gagaatgggagatgaagttgg	gttcacctagcactttgtgg
***Antxr1***	335	D	catatggctgtcaacagcaagg	gagtgtcggttaaggagaag
***Antxr1***	157	D	gacgatctccaaagattcgg	gtaggactctgtggctgatg
***Antxr1***	363	C	gttctgccaggaggagacac	gcagatggtggatggttcag
***Antxr1***	490	C	aggctctccaaggcattatcc	ccatcatcgtcttcttcctcac
***Antxr1***	489	C	gctgctctggtggttctg	gtgttgttcaggggatacttg
***Ap2a1***	510	C	agaacgctatcctctttgagacc	ggacgtcatcacggttgg
***Ap2a1***	543	C	ggcttttgctgcagacattc	aggttgggctcactgtcatag
***Ap2a1***	442	C	gttgtcggtgcgcttcc	catataccagggcaagtccag
***Ap2a1***	531	C	ctatgtgagcgaggaggtgtgg	cggctgtcatctagggcactg
***Ap2a1***	351	C	agcgggagtcgtccatcttg	gagctgggtcagccaacaaag
***Pak4***	503	C	aacacatacccacgggctgac	cgcatgatcaccacctcattg
***Pak4***	477	C	gtacgcgggcacagagttc	ccgacatgttctcaaattcgtc
***Pak4***	459	C	aggatggggctctcactctg	cattaggggccatggtatgtg
***Pak4***	412	C	caagcagcaaagacgtgaaactg	atagggaaggcgggagatgag
***Pak4***	447	C	ctgtccgacttcgggttttgtg	gtagccaggctctttggttcaagac

To perform the PCR reactions with genomic DNA templates, master mixes were made for each amplicon (or multiplexed amplicons) and 10 μl of the mix were added to wells 1–93, which contained the following components: 10 ng dried DNA template, 1× PCR buffer (20 mM Tris-HC1, pH 8.0, 500 mM KCl; Invitrogen), 40 ng of each primer (Sigma-Genosys, The Woodlands, TX), 1.5 mM MgCl_2_, 0.4 mM dNTPs (Invitrogen) and 0.5 U of Platinum *Taq *DNA polymerase (Invitrogen). To perform the PCR reactions with cDNA templates, master mixes were made for each amplicon (or multiplexed amplicons) and 20 μl of the mix was added to wells 1–93, which contained the following components: 0.8 μl dried cDNA template, 1× PCR buffer (same as for DNA templates), 1.75 mM MgCl_2_, 0.2 mM of each dNTP, 0.2 mM of each primer (Integrated DNA Technologies, Coralville, IA), and 0.1 μl (1 U) of Platinum *Taq *DNA polymerase (Invitrogen). Master mix with primers specific for the known ENU-induced mutation was added to the dried template in well 94 of each plate. PCR amplification was conducted in either Peltier Thermal Cycler-200 units (MJ Research/Bio-Rad Laboratories, Waltham, MA), a DNA Engine Tetrad 2 (MJ Research/Bio-Rad Laboratories), or Eppendorf Mastercycler units (Brinkmann/Eppendorf, Westbury, NY). The standard cycling conditions used were as follows (with the annealing temperature and sometimes the number of cycles varying with certain amplicons): an initial 94°C incubation for 3 min; followed by 30 cycles of 94°C for 30 sec, 60°C annealing for 30 sec, and 72°C for 45 sec; and a final incubation at 72°C for 7 min. After completion of PCR, the reactions were denatured and reannealed to allow the formation of any heteroduplexes and the plates were stored at -20°C until they were screened by TGCE for the presence of heteroduplexes.

### Mutation screening PCR amplicons by TGCE with the SCE9612

Prior to TGCE analysis of the PCR products, additional positive-control samples (for TGCE) were added to wells 95 (H11) and 96 (H12) of each plate. Well 95 contained DNA for the same known ENU-induced mutation as well 94. However, the difference for well 95 is that PCR of the DNA and heteroduplex formation of the PCR product were previously performed in bulk, and an aliquot of the sample was previously shown to display the mutation (heteroduplex) by TGCE analysis. Well 96 contained DNA from a second known ENU-induced mutation in which PCR and heteroduplex formation were also previously performed in bulk.

Heteroduplexed PCR products were analyzed by TGCE in the SpectruMedix (State College, PA) SCE9612 Reveal Mutation Detection System. The TGCE reactions were typically conducted using a single 50–60°C temperature gradient, 5-Kv injection voltage, and 30-sec injection time. Adjustments in the injection voltage and injection time were occasionally necessary for screening the cDNA templates due to the variability in the amount of RT-PCR products in multiplexed reactions, which is inherent in the differences in the expression levels for different genes. Moreover, in some instances, dilutions (1:20–1:40) of RT-PCR products were also required for optimal TGCE analysis of cDNA templates amplified from genes that are highly expressed.

Separations of heteroduplexes from homoduplexes were achieved with the Reveal high-resolution matrix (SpectruMedix MREV-HR-225-001v3). The DNA fragments in 1× running buffer (diluted 5× buffer; SpectruMedix BRUR-500-003) were detected in the SCE9612 by measuring laser-induced fluorescence of the ethidium bromide-stained DNA. Raw-image data files were generated by the SpectruMedix Checkmate software, and subsequently processed by Revelation 2.4 mutation analysis software that produced baseline-corrected single-color traces for every capillary and automatically compared electropherograms from experimental and control samples. Identification of samples containing heteroduplexes was performed by both Revelation 2.4 software analysis and visual inspection of the electropherograms.

### DNA sequence analysis

Direct DNA sequencing of the PCR-amplified gene fragments was performed: (1) on amplicons from CMMB samples yielding heteroduplexes (as identified by TGCE analysis) in order to identify the ENU-induced point mutation; (2) on amplicons from sperm DNA of CMMB mice prior to ICSI; (3) and on DNA from tail biopsies or ear punches of mice recovered by ICSI for genotyping. Genomic DNA was extracted from sperm with the HotSHOT method [49] using 5–10 μ1 of the same sperm sample (in NIM and 8% PVP, see below) that was used for ICSI. Typical PCR reactions included: 1× PCR buffer, 2.27 mM MgCl_2_, 0.52 mM dNTPs, 260 ng of each primer, 40 ng of DNA template, and 5 units of Platinum *Taq *DNA polymerase (Invitrogen) in a total reaction volume of 100 μl. Typical PCR conditions were: 94°C for 5 min; 29 cycles of 94°C for 30 sec, the optimal annealing temperature for each primer pair for 45 sec, 72°C for 30 sec; 72°C for 2 min; and 4°C hold. The PCR products were purified with the QIAquick PCR Purification Kit (Qiagen) and DNA was eluted from the columns with 30 μl of elution buffer. The purified DNA and primers were submitted to the DNA sequencing core at the University of Tennessee, Knoxville, where DNA was labeled and fluorescent automated DNA sequencing was performed on an ABI PRISM 3100 Genetic Analyzer (Applied Biosystems, Foster City, CA) according to standard protocols.

### Mice used for ICSI

Mice were obtained at 5 weeks of age from the following sources: B6D2F1 (C57BL/6 × DBA/2) from Taconic (Germantown, NY) and Harlan (Indianapolis, IN), and C57BL/6J from The Jackson Laboratory (Bar Harbor, ME). The mice were fed *ad libitum *with a standard diet and maintained in a temperature and light-controlled room (22°C, 14L:10D) in ventilated caging and racks (Thoren, Hazleton, PA) with automated watering (Edstrom Industries, Waterford, WI) in a specific-pathogen free (SPF) environment.

### Oocyte collection for ICSI

Mice (7–12 weeks of age) were superovulated with IP injections of 5 IU PMS (Calbiochem, San Diego, CA) and 5 IU HCG (Sigma-Aldrich) given 48 hours apart. Oviducts were removed 15 hours (B6D2F1) or 12 hours (C57BL/6J) after HCG injection and placed in PBS in a petri dish. The cumulus-oocyte mass was released from the ampulla of the oviduct by rupturing the oviduct with a pair of tissue forceps into 0.1% bovine testicular hyaluronidase (Sigma-Aldrich) in M2 medium (Specialty Media, Phillipsburg, NJ) to disperse the cumulus cells. The oviduct was discarded and the oocytes collected and washed 3× in M2 medium and placed in Fertilization Medium (Cooper Surgical, Trumbull, CT) in a 37°C incubator with 5% CO_2 _in air and allowed to "rest" for 1 hour before ICSI.

### Sperm preparation for ICSI

One straw containing the sperm from a mouse in the CMMB with the desired ENU-induced mutation was removed from liquid nitrogen and thawed immediately in a 37°C water bath. The straw was sprayed with 70% ethanol and aseptically cut on both ends and the contents collected in a 1.5 ml microcentrifuge tube. After centrifugation for 10–15 seconds at high speed, the pelleted sperm were then washed in 1 ml NIM medium (nucleus isolation medium; 125 mM KCl, 2.6 mM NaCl, 7.8 mM NaH_2_PO_4_, 1.4 mM KH_2_PO_4_, 3.0 mM EDTA disodium salt in ultrapure water, pH adjusted to 7.2, filtered and autoclaved) and centrifuged again for a few seconds. The sperm pellet was then resuspended in 300 μl of NIM medium, pipeted vigorously (to aid in removing the tails from the sperm heads), and 15 μl aliquots were distributed into cryovials and refrozen by placing and storing the vials in the vapor phase of liquid nitrogen in a liquid nitrogen freezer. One aliquot was used for ICSI per day.

### ICSI procedure

ICSI was carried out essentially as described by Kimura and Yanagimachi [35] with a few modifications. Injection needles (ID 6 mm, 25° bevel) were purchased from Humagen (Charlottesville, VA). Holding pipets (25° bevel) were purchased from Eppendorf (Westbury, NY). A 15 μl aliquot of the sperm sample in NIM medium was mixed 1:1 with NIM medium containing 16% polyvinyl pyrrolidone (PVP, M_r _360 kDa), resulting in sperm in NIM with 8% PVP for ICSI. Two 7 μl drops of the sperm sample were placed on the injection plate (the cover of a plastic 100 mm Optilux petri dish, BD Falcon, No. 351005), together with two 7 μl drops of NIM with 8% PVP (to condition the injection needle) and two 7 μl drops of M2 medium (where oocytes were placed and ICSI occurred), which were all overlaid with Mineral Oil containing Vitamin E (Walgreens, Oak Ridge, TN). ICSI was performed using Eppendorf Micromanipulators (TransferMan NK2) with a piezo-electric actuator (Piezo Impact Micro-Manipulator, PMM-150FU System; PrimeTech, Ibaraki, Japan). A single sperm head (without the tail) was collected and injected immediately into an oocyte. The speed and intensity settings on the PMM operation box (OP-15) for zona coring were 5–6 and 5, respectively. The speed and intensity of oolema penetration were both 1–2. ICSI was performed 1–4 hours after oocyte collection. Sperm-injected oocytes were transferred into another dish of M2 Medium at room temperature to allow them to "rest" for approximately 20 min before being transferred into pre-equilibrated Cleavage Medium (Cooper Surgical) for several hours at 37°C, 5% CO_2_. Cleavage Medium was changed every hour prior to overnight culture.

### Preimplantation culture of injected ova

Following ICSI, the injected ova were cultured overnight to the 2-cell stage. Groups of 15–20 oocytes were transferred from Cleavage Medium to 25-μl drops of KSOM Medium (Specialty Media) that were overlaid with Embryo Tested Mineral Oil (Sigma-Aldrich) and pre-equilibrated at 37°C and 5% CO_2 _in 60 mm plastic culture dishes (BD Falcon, No. 353002).

### Embryo transfer

After overnight culture, the 2-cell embryos were transferred into the oviducts of pseudopregnant ICR (Harlan) and B6D2F1 mice. The females were naturally mated to vasectomized ICR males. Following analgesia (Buprenex) and anesthesia (Avertin), the oviducts of pseudopregnant females were exposed through a small incision in the skin and body wall. The embryos (15–40) were transferred unilaterally into one ampulla with a glass capillary through a small opening in the oviduct between the ovary and ampulla made with the tip of a 30-gauge needle. The body wall was closed with a sterile absorbable suture and the skin was closed with a wound clip. Mice were permitted to recover from surgery in a pre-warmed cage.

### Genotyping mutant mice rederived by ICSI

The wild-type and heterozygous mutant G_2 _mice recovered by ICSI were genotyped by the same PCR and TGCE strategy (described above) originally used to identify the mutation in the DNA samples. The homozygous wild-type, heterozygous, and homozygous mutant offspring produced from the intercrosses of heterozygous mutant mice were genotyped either by TGCE or DNA sequencing of PCR products. For large numbers of mice, TGCE was used to identify heterozygous mice from homozygous mice. Since TGCE cannot distinguish between homozygous mutant and homozygous wild-type mice, the PCR products from all homozygous mice were then mixed with an equal volume of PCR product from a wild-type C57BL/6J control mouse and the TGCE was repeated, which then resulted in the identification of homozygous mutants (heteroduplexes detected) from homozygous wild-types (no heteroduplexes detected). Alternatively, smaller numbers of mice were genotyped by direct DNA sequencing of PCR products, as described above.

## Authors' contributions

Project conception and design were by EJM, CTC, MLK, EMR, and DKJ. Construction of the CMMB, including ENU injections, animal husbandry, tissue harvesting, and sperm and tissue cryopreservation were by EJM, PEB, KTC, DJC, LLE, AWG, KJH, REO, IP, GDS, SGS, EMR, and DKJ. Preparation and quantification of DNA were by EJM, CMF, AWG, LAH, and AMW. Preparation and quantification of RNA and cDNA were by MLK, DJC, and REO. Screening the DNA and cDNA templates for ENU-induced mutations by TGCE was by CTC, PEB, LLE, ZYG, LAH, MKK, ZL, and AMW. DNA sequence analysis was by EJM, CTC, LAH, MLK, LLE, and AMW. Recovery of mutant mice from cryopreserved sperm, animal husbandry, and genotyping were by EJM, CMF, KJH, and AMW. The manuscript was drafted by EJM, CTC, and MLK. All authors made improvements to the draft of the manuscript and approved the final version.
